# Functional effects of variation in transcription factor binding highlight long-range gene regulation by epromoters

**DOI:** 10.1093/nar/gkaa123

**Published:** 2020-02-29

**Authors:** Joanna Mitchelmore, Nastasiya F Grinberg, Chris Wallace, Mikhail Spivakov

**Affiliations:** 1 Nuclear Dynamics Programme, Babraham Institute, Babraham Research Campus, Cambridge CB22 3AT, UK; 2 Cambridge Institute of Therapeutic Immunology & Infectious Disease (CITIID), University of Cambridge, Cambridge Biomedical Campus, Cambridge CB2 0AW, UK; 3 MRC Biostatistics Unit, University of Cambridge, Cambridge Biomedical Campus, Cambridge CB2 0SR, UK; 4 MRC London Institute of Medical Sciences, Du Cane Road, London W12 0NN, UK; 5 Institute of Clinical Sciences, Faculty of Medicine, Imperial College, Du Cane Road, London W12 0NN, UK

## Abstract

Identifying DNA cis-regulatory modules (CRMs) that control the expression of specific genes is crucial for deciphering the logic of transcriptional control. Natural genetic variation can point to the possible gene regulatory function of specific sequences through their allelic associations with gene expression. However, comprehensive identification of causal regulatory sequences in brute-force association testing without incorporating prior knowledge is challenging due to limited statistical power and effects of linkage disequilibrium. Sequence variants affecting transcription factor (TF) binding at CRMs have a strong potential to influence gene regulatory function, which provides a motivation for prioritizing such variants in association testing. Here, we generate an atlas of CRMs showing predicted allelic variation in TF binding affinity in human lymphoblastoid cell lines and test their association with the expression of their putative target genes inferred from Promoter Capture Hi-C and immediate linear proximity. We reveal >1300 CRM TF-binding variants associated with target gene expression, the majority of them undetected with standard association testing. A large proportion of CRMs showing associations with the expression of genes they contact in 3D localize to the promoter regions of other genes, supporting the notion of ‘epromoters’: dual-action CRMs with promoter and distal enhancer activity.

## INTRODUCTION

Identifying DNA cis-regulatory modules (CRMs) that control the expression of specific genes is crucial for deciphering the logic of transcriptional control and its aberrations. Advances of the last decade have made it possible to predict active CRMs based on chromatin features (1,2) and detect the binding of dozens of transcription factors (TFs) to these regions (3,4). However, deletion of known or predicted CRMs often shows no observable phenotype, suggesting that some CRMs either lack appreciable gene regulatory function or are efficiently buffered by other sequences, at least under normal conditions (5–9). In addition, the sequence, chromatin state and genomic location of CRMs do not immediately provide information on their target genes (10). Therefore, evidence from complementary approaches is required to establish the function of specific CRMs in transcriptional control.

Natural genetic variation can theoretically provide a direct indication of gene regulatory function by revealing the allelic associations between specific variants and gene expression (11,12). While expression quantitative trait loci (eQTLs) identified this way have provided important insights into gene control and the mechanisms of specific diseases (13,14), a number of challenges hamper comprehensive detection of functional sequences in ‘brute-force’ eQTL testing (15,16). In particular, the immense search space leads to a heavy multiple testing burden resulting in reduced sensitivity. This problem is typically mitigated in part by testing for ‘cis-eQTLs’ separately within a limited distance window (∼100 kb); this distance range is, however, an order of magnitude shorter than that of known distal CRM activity (17–19). In addition, correlation structure arising from linkage disequilibrium (LD) requires disentangling causal from spurious associations, which is particularly challenging in the likely scenario, whereby multiple functional variants with modest effects co-exist within the same LD block (20). These challenges provide a strong motivation for incorporating prior knowledge into association testing for identifying causal regulatory variants.

The recruitment of TFs to CRMs plays a key role in the regulatory function of these elements (21,22), and mutations leading to perturbed TF binding are known to underpin developmental abnormalities and disease susceptibility (18,23,24). Therefore, sequence variation affecting TF binding affinity at CRMs has a strong potential to have causal influence on their function and can provide insights into the logic of gene control. Variation in TF binding across multiple individuals has been assessed directly for several TFs (25–30), but high resource requirements of these analyses limit the number of TFs and individuals profiled this way. Alternatively, the effects of local sequence variation on TF binding can be predicted, at least in part, based on prior information regarding the TFs’ DNA binding preferences. The representation of such preferences in the form of position weight matrices (PWMs) (31) has proven particularly useful, as it provides a quantitative measure of how much a given sequence substitution is likely to perturb TF binding consensus. Consistent with this, we and others have previously shown that the specificity of TF binding preferences to a given motif position correlates with the functional constraint of the underlying DNA sequences, both within and across species (32–34). Classic PWM-based approaches to TF binding prediction focused on identifying short sequences showing a non-random fit to the PWM model compared with background (35,36). More recently, biophysical modelling of TF binding affinity (37,38) has provided a natural framework to extend this analysis by integrating over all PWM match signals within a DNA region (39,40), including those from lower affinity sites that are a known feature of many functional CRMs (41–43).

Long-range CRMs such as gene enhancers commonly act on their target promoters through DNA looping interactions (44,45). Therefore, information on 3D chromosomal organization enables predicting the putative target genes of these elements (46,47) and thus has the potential to significantly improve the functional interpretation of regulatory variation. Approaches that couple chromosome conformation capture with target sequence enrichment such as Promoter Capture Hi-C (PCHi-C) (48–50) are particularly useful in this regard, as they make it possible to detect regulatory interactions globally and at high resolution with reasonable amounts of sequencing (51–59).

Here, we integrate TF binding profiles in a human lymphoblastoid cell line (LCL) (4) with patterns of natural sequence variation (60) to generate an atlas of CRMs predicted to show significant TF binding variability across LCLs derived from multiple individuals. We delineate the putative target genes of these CRMs from their interactions with gene promoters based on PCHi-C and linear proximity (49,61) and test for associations between the CRMs’ TF binding affinity and target gene expression using transcriptomics data for hundreds of LCLs (62). Prioritizing CRMs that show predicted variation in TF binding affinity based on a biophysical model (39,40) makes it feasible to perform association analysis in a manner that accounts for multiple variants affecting the binding of the same TF, as well as for multiple CRMs targeting the same gene. Using this approach, we reveal >1300 CRM variants associated with expression of specific genes, the majority of them undetected with conventional eQTL testing at a standard false discovery rate (FDR) threshold. We find that a large proportion of CRMs showing associations with the expression of distal genes localize in the immediate vicinity of the TSSs of other genes and connect to their targets via DNA looping interactions, suggesting their role as ‘epromoters’: the recently identified dual-action regulatory regions with promoter and distal enhancer activity (63–65).

## MATERIALS AND METHODS

### CRM definition

ChIP-seq narrow peak files for 52 TFs in GM12878 were downloaded from the UCSC ENCODE portal (4). Where multiple datasets were available for the same TF, the intersect of the ChIP-seq peaks was taken for all TFs except ERG1, for which we took the union of the two datasets available, since one of them had substantially fewer peaks than the other. CRMs were defined by taking the union of the peaks for the 52 TFs with a minimum overlap of one base pair.

### Detection of TF binding affinity variants

Variant calls for 359 LCLs of European ancestry (CEU, TSI, FIN, GBR and IBS) that overlapped with the CRMs defined earlier were downloaded from the 1000 Genomes Project (release Phase 3; 20130502) (60). Multi-allelic variants and variants with a minor allele frequency <5% were removed. Unique haplotypes (i.e. unique combinations of single-nucleotide polymorphisms [SNPs] and insertions/deletions [indels]) were identified across the 359 LCL individuals for each CRM. The GRCh37 genomic sequence for each CRM (accessed using the Bioconductor package BSGenome, https://doi.org/10.18129/B9.bioc.BSgenome) was then patched to create the sequence for each unique haplotype.

For each TF detected as bound at a given CRM in GM12878 (based on ChIP-seq data), we computed the affinity for each haplotype and each PWM for this TF available from ENCODE (66). The library of ENCODE motifs was imported from the R package atSNP (67), and 41/52 TFs for which there was an exact match between TF name and motif name were taken forward to the analysis. TF affinities were computed using the TRAP biophysical model (39) as implemented in the R package tRap (https://github.com/matthuska/tRap). Default parameters were used, with the exception of setting pseudocount to zero, since we were using frequency as opposed to count matrices. We chose TRAP over approaches based on individual motif hits, as it naturally incorporates the effects of multiple low-affinity sites and multiple variants per CRM.

CRM binding affinities were normalized using a method proposed by Manke *et al.* (40), such that changes in them could be compared between different PWMs. Briefly, CRM affinities are converted to statistical scores (*A*) representing the probability of observing a given or higher affinity for a given TF in the background sequence (note that lower values of *A* therefore reflect higher affinities). Binding affinities are parameterized using the extreme value distribution whose parameters are estimated for a range of background sequences encompassing the lengths of all CRMs (40, 100, 200, 250, 300, 400, 500, 800, 1000, 2000 and 3000) using the fit.gev function in the R package tRap. CRMs not bound by a given TF are cut/extended to the required length and used as background sequences.

For all CRM TF/PWM combinations with *A* < 0.1 in the highest affinity allele of GM12878, we computed the log fold change in affinity between all observed haplotypes and the highest affinity allele of GM12878:

$$\begin{equation*}{\rm{log\,FCA}} = {\rm{log}}_{10}({A_{\rm{ALT}}})-{\rm{log}}_{10}({\rm{min}}({A_{{\rm{GM}}12878}})),\end{equation*}$$

where min(*A*_GM12878_) is the normalized affinity of the highest affinity allele in GM12878 cells and *A*_ALT_ is the normalized affinity of the alternative haplotype. For instances where *A*_ALT_ or *A*_GM12878_ for a given PWM was zero, the lowest observed non-zero normalized affinity for that PWM across all CRMs was used instead. The log FCA values for multiple PWMs of the same TF were then combined by taking the median. Overall, this approach produced a single log FCA for each TF binding affinity haplotype at each CRM. We shall refer to this quantity as the ‘log ratio’ in the ‘Results’ section.

### DeepSea analysis

For all SNPs at CRMs, DeepSea (68) predictions were obtained using the online tool (http://deepsea.princeton.edu/job/analysis/create) with the SNPs in VCF files provided as input, in seven batches. Since the predictions of log fold change in signal generated by DeepSea can be noisy when probabilities are small, we used ‘chromatin feature probability differences’ (.diff files) as robust predictors. DeepSea predictions available for 33/41 TFs analysed in our study, as well as for DNase-seq signals, were used for comparison with our biophysical model predictions of TF binding affinity effects at SNP level.

### DNase I sensitivity QTL analysis

The DNase I sensitivity QTL (dsQTL) dataset from (69) lists significant associations between normalized DNase-seq read depth (binned in 100 bp non-overlapping windows) and the genotypes of SNPs/indels within 1 kb of the DNase hypersensitivity sites (DHS) in 70 Yoruban LCLs. We downloaded this dataset from Gene Expression Omnibus (accession number GSE31388), and converted it to GRCh37 using liftOver (70). For all CRMs with a predicted log FCA > 0 for at least one TF, the individual effect of all SNPs at the CRM on TF affinity was calculated. CRMs were then filtered for those where the SNP causing the largest change in TF affinity (‘driver SNP’) had a minor allele frequency (MAF) below 0.05 in the 70 individuals from (69). We then counted the number of overlaps between these CRMs and the 100 bp DHS windows (minimum overlap 1 bp), repeating this for CRMs filtered according to successively larger log FCA thresholds. To estimate expected overlap, for each threshold, we randomly sampled a control set of CRMs 1000 times, matching the sample size and ‘driver’ SNP allele frequency distribution to the test set at a given threshold, and overlapped this set with DNase HS windows in the same way as the test set.

### Comparison with ATAC-QTLs

ATAC-QTLs from (71) detected in at least two populations at *P* < 0.005 were used for analysis. For all SNPs at CRMs with a predicted log FCA > 0, we calculated the proportion of overlapping ATAC-QTLs over the exceeding thresholds of the maximum log FCA across all analysed TFs for each SNP. To estimate the expected overlap, we randomly sampled a control set of CRMs 100 times, matching the sample size and minor allele frequency distribution to those in the test set at a given threshold.

### Comparison with MPRA data

MPRA results were downloaded from (72). The effects of SNPs on reporter expression (combined log_2_ skew over two LCLs tested) were used for comparison with their maximum predicted effects on TF binding affinity obtained from the biophysical model in our study.

### Linking of CRMs with target genes

PCHi-C data for GM12878 were obtained from Mifsud *et al.* (49). Significant interactions were re-called at a *HindIII* restriction fragment level using the CHiCAGO pipeline (61), with a CHiCAGO score cut-off of 5 (CHiCAGO scores correspond to soft-thresholded, log-weighted *P*-values against the background model). Baits were annotated for transcriptional start sites (TSSs) using the bioMart package in R (73) based on Ensembl TSS data for GRCh37 reference assembly. Baits containing TSSs for more than one gene were excluded (4178 out of 22 076), leaving 17 898 baits in the analysis. CRMs were assigned to target promoters by overlapping with the promoter-interacting regions of significant interactions (‘distal’ CRMs). Restriction fragments immediately flanking the promoter fragment are excluded from PCHi-C analysis, creating a ‘blind window’. Therefore, we additionally called ‘proximal’ CRMs using a window-based approach, assigning all CRMs located within 9 kb of the midpoint of the promoter-containing fragment to the respective promoter.

### Gene expression data processing

We downloaded PEER-normalized (74) gene-level RPKMs for 359 EUR LCLs profiled in the GEUVADIS project (62) from ArrayExpress (75) (accession E-GEUV-3). The data were filtered to expressed genes by removing genes with zero read counts in >50% of samples. For expression association testing by linear regression, the PEER-normalized residuals for each gene were further rank-transformed to standard normal distribution, using the rntransform function in the R package GenABEL (76).

### Association between TF binding affinity variants and gene expression: thresholded approach

In this approach, we classified each predicted TF binding affinity CRM haplotype as either ‘high’ or ‘low’ affinity based on a threshold. In some instances, however, using a hard threshold to classify alleles can result in alleles with very similar log fold affinity changes being differentially classified, which can obscure true affinity–expression associations. To avoid this, we used a dynamic thresholding approach, where for each affinity variant we set the threshold log FCA_0_ to 80% of the value of the 85th percentile of all log FCA values less than or equal to the hard threshold of −0.3. All alleles with log FCA ≤ log FCA_0_ were taken as low affinity. Alleles with either log FCA > log FCA_0_/4 (for log FCA_0_/4 > −0.3) or log FCA > −0.3 were taken as high affinity. Note that this resulted in some alleles classified as neither high nor low affinity. Individuals containing at least one unclassified allele for a given TF/CRM were excluded from the testing for the respective association (the number of individuals tested for each association is listed in Supplementary Table S1).

A regression model was then fitted using TF binding affinity CRM haplotypes as predictors of the expression level of their target genes (presented in terms of normalized PEER residuals). Suppose that a gene is targeted by *K* predicted TF affinity CRM variants, denoted as *X* = (*X*_1_, *X*_2_, …, *X_K_*), which are encoded as the number of copies of the low-affinity allele carried by each individual. The regression model is fitted as follows:

$$\begin{equation*}E[Y] = {\beta _0} + {\beta _1}{X_1} + {\beta _2}{X_2} + \cdots + {\beta _K}{X_K},\end{equation*}$$

where *E*[*Y*] is the expected value of the normalized PEER residuals *Y*. Where multiple predicted TF affinity CRM variants targeting a given gene were in perfect correlation (|*β*| > 0.99), they were collapsed into a single predictor.

ANOVA was used to test the overall significance of each regression model, with multiple testing correction performed on the gene-level *P*-values by FDR estimation. For genes showing significant associations at 10% FDR in models with multiple TF binding affinity variants as predictors, *t*-tests were performed to identify variants with regression coefficients significantly different from zero. Variants with unadjusted coefficient-level *P*-values <0.05 were taken to be significantly associated with target gene expression, conditional on significant gene-level association at 10% FDR.

### Association between TF binding affinity variants and gene expression: threshold-free approach

In this approach, we performed multiple regression using PEER expression residuals for each gene as the response variable, this time using the sum of log FCA across both alleles for each individual for each TF affinity CRM variant as predictors instead of thresholded CRM haplotypes. For each gene, all distal and proximal CRMs with log FCA > 0 were included. As with the thresholded approach, ANOVA was used to test the significance of each gene model, and genes showing associations at 10% FDR were considered significant.

Due to high collinearity among the predicted affinity changes, to identify specific CRM variants significantly associated with target gene expression we used elastic net regression for each significantly associated gene (*λ*_2_ = 0.5). The significance of each predictor as it entered the model was then tested using a method by Lockhart *et al.* (77) and implemented in the R package covTest (https://cran.r-project.org/src/contrib/Archive/covTest/covTest_1.02.tar.gz). Variants that entered the model with *P* < 0.05 and remained in the model were taken as significant.

### eQTL fine mapping

We fine-mapped eQTL causal variants in the LCL expression data within a window of ±200 kb of each CRM, using a Bayesian stochastic search fine-mapping method that allows for multiple causal variants, GUESSFM (https://github.com/chr1swallace/GUESSFM) (78). This requires a prior on the number of causal variants per region, which we set as Bin(*n*, 2/*n*) where *n* is the number of variants in the fine-mapping window. This setting gives a prior expectation of two causal variants per region but allows all values from 0 to *n*. We visually checked traces to ensure the Markov chain Monte Carlo (MCMC) samples had converged. Raw GUESSFM data have been uploaded to the Open Science Framework (OSF; https://osf.io/e5vsh/).

To estimate the proportion of possibly causal eQTLs identified by GUESSFM (marginal posterior probability of inclusion $[{\rm mppi}]\gg 0.001$) among the TF binding affinity variants showing the strongest eQTL signal per CRM (‘test SNPs’), we compared it with the same proportion obtained for ‘random SNPs’. The ‘random SNPs’ were sampled from the same ±200 kb windows around CRMs, matching the distribution of their minor allele frequencies to that across the ‘test SNPs’.

### Causal variant colocalization analysis

An association between an epromoter variant and the expression of both a proximal and a distal gene may indicate that this variant is causal for the expression of both genes. However, the same association may arise from distinct causal variants for each gene that are in LD with each other and are tagged by the same epromoter variant. To differentiate between these situations, we used the Bayesian colocalization technique coloc (79). Coloc evaluates the posterior probabilities of five mutually exclusive hypotheses: no association of any variant in the region with either trait (H0), association with first trait but not the second (H1), association with second trait but not the first (H2), two separate causal variants (H3) and finally a unique shared causal variant (H4). Coloc assumes at most one causal variant per locus. To mitigate this limitation, where there was evidence for multiple causal variants, we tested for colocalization between all pairs of signals for each gene by conditioning out the other signals. Coloc has also been originally designed for testing two sets of associations measured on different individuals. Therefore, before running it on the data measured in the same individuals (i.e. the expression of the proximal and distal gene across the 359 CEU LCLs), we confirmed by simulation that for a quantitative trait the results appear robust to correlated errors (Supplementary Figure S1).

## RESULTS

### An atlas of CRMs with predicted variation in TF binding affinity in LCLs

**Figure 1.**
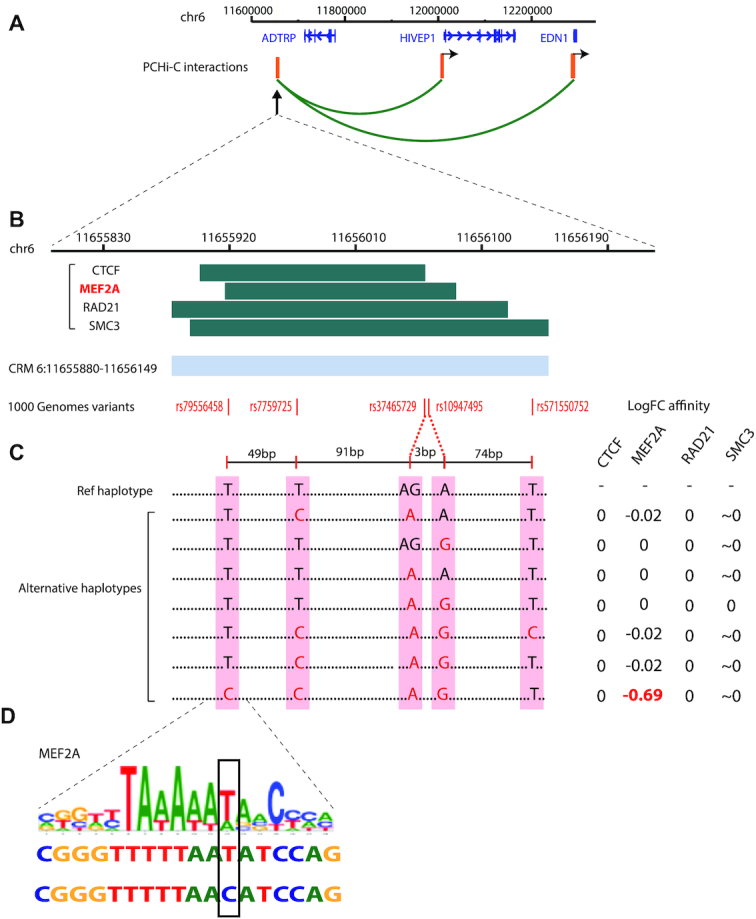
Variation in TF binding affinity at a distal CRM. (**A**) Chromosomal interactions (green arcs) of a distal CRM on chromosome 6 (left) with two downstream genes in the LCL GM12878 detected by PCHi-C (49,61). *HindIII* restriction fragments containing the CRM (left) and baited promoters (two fragments on the right) are shown in orange. (**B**) Top: the ChIP-seq peaks from ENCODE (4) detected at this CRM (dark green) and used to define its boundaries (light blue). Bottom: the positions of 1000 Genomes Project SNPs within the CRM (red). (**C**) CRM haplotypes detected in 1000 Genomes Project (left) and changes in the affinity for the bound TFs at them with respect to the reference haplotype estimated using the biophysical model (39). (**D**) The MEF2A motif instance underlying the highest change in the affinity of this TF at the CRM. Top: motif logo; bottom: the reference and alternative haplotypes for this instance.

We used the ChIP-seq binding profiles of 52 TFs profiled by the ENCODE project (4) in GM12878 LCL to define 128 766 CRMs in these cells, merging across overlapping ChIP regions for multiple TFs (Figure 1). Just over half (55%) of CRMs defined this way were bound by more than a single TF. For 41/52 TFs with known PWMs, we then used a biophysical model (39) to estimate their binding affinity to each allele of each CRM in GM12878, pooling information across multiple PWMs for the same TF where available (see ‘Materials and Methods’ section). To enable the comparison of binding affinities between different TFs, we expressed them relative to the respective ‘background’ affinities using an approach based on the generalized extreme value distribution (40) (see ‘Materials and Methods’ section for details).

We next asked how natural genetic variation at CRMs affects their TF binding affinity. For this, we took advantage of the genotypes of an additional 358 LCLs also derived from European-ancestry individuals that are available from the 1000 Genomes Project (60). These LCLs showed sequence variation at 98 918 (79%) of the CRMs relative to GM12878. We then calculated a TF affinity log-ratio between each alternative haplotype and the highest-affinity haplotype of GM12878 (Figure 1; see ‘Materials and Methods’ section). SNP-level effects on TF affinity predicted by the biophysical model showed a significant correlation with those predicted by a deep learning algorithm DeepSea (68) trained on epigenomic data across tissues (*r* = 0.36, corr test *P* < 2.2e−16, Supplementary Figure S2A). Overall, 38 804 CRMs had one or more alternative haplotypes with predicted changes in binding affinity for at least one TF (affinity log ratios ranging between −12.9 and 13.17). We have made the full atlas of TF-binding CRM variants publicly available at https://osf.io/fa4u7.

### TF-binding variants are enriched for associations with chromatin accessibility and effects on reporter gene expression

**Figure 2.**
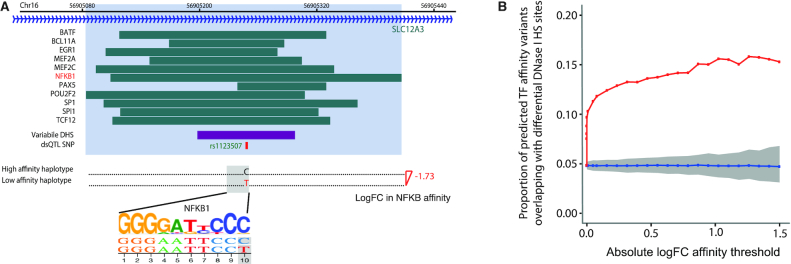
TF binding affinity variants are enriched at regions showing variation in DNase I hypersensitivity. (**A**) An example of a CRM with a dsQTL affecting TF binding affinity. Top: the locations of ChIP-seq peaks from ENCODE (4) detected at this CRM (dark green) shown alongside the variable DNase I hypersensitivity site [DHS; detected by DNase-seq (69)] and its cognate dsQTL SNP (69). CRM boundaries are coloured in light blue. Bottom: the predicted effect of the dsQTL SNP on NFKB binding affinity estimated by the biophysical model (39). (**B**) The proportion of CRMs with log-fold affinity changes over a range of thresholds that overlap with differential DHS (69) are depicted by red squares. CRMs were filtered to those where the SNP driving the affinity change has a MAF > 5% in the 70 YRI individuals. The mean proportion of randomly sampled CRMs that overlap with differential DHS across 1000 permutations is shown in blue, with the grey ribbon showing the 90% range. For each threshold, the control sets of CRMs were matched in sample size and the ‘driver’ SNP's allele frequency distribution to those of the predicted affinity variants over the corresponding threshold.

TF binding is known to be associated with increased chromatin accessibility. Consistent with this, variant effects on TF affinity predicted by the biophysical model correlated with DeepSea-predicted effects on DNase I signal (*r* = 0.33, corr test *P* < 2.2e−16, Supplementary Figure S2B). To validate these effects more directly, we took advantage of a published study (69) that profiled chromatin accessibility across 70 LCLs using DNase-seq and identified ∼9000 significant associations between DNase-seq signal and genotype (dsQTLs). If our predicted TF affinity variants reflected real changes in binding affinity, we would expect them to be enriched at regions of differential chromatin accessibility (see Figure 2A for an example). To verify this, we quantified enrichment of differential chromatin accessibility at sets of CRMs showing predicted TF affinity variation above successively larger thresholds. As can be seen from Figure 2B, CRMs with non-zero differences in TF binding affinity across LCLs showed a significant enrichment at differential DNase I sensitivity regions compared with a matched random set of CRMs (permutation test *P* < 0.001, see ‘Materials and Methods’ section for details). Moreover, this enrichment increased with the magnitude of the predicted affinity change (Figure 2B).

ATAC-seq provides another readout of chromatin accessibility. Consistent with the findings from DNase-seq analysis, we observed that the magnitude of variant effects on TF affinity positively associated with an enrichment for ATAC-QTLs from a recent study using a much larger cohort of LCLs across populations (71) (Supplementary Figure S3).

Finally, we assessed the effects of TF-binding variants on reporter gene expression using data from a massively parallel reporter assay in LCLs (MPRA) (72), which included results for 1519 variants mapping to the CRMs from our study. Variant effects on reporter activity showed a significant correlation with those on TF affinity (*r* = 0.11, corr test *P* = 0.005, Supplementary Figure S4).

Jointly, these results provide evidence that our approach adequately predicts functionally relevant variant effects on TF binding.

### Variation in TF binding affinity at CRMs associates with target gene expression

**Figure 3.**
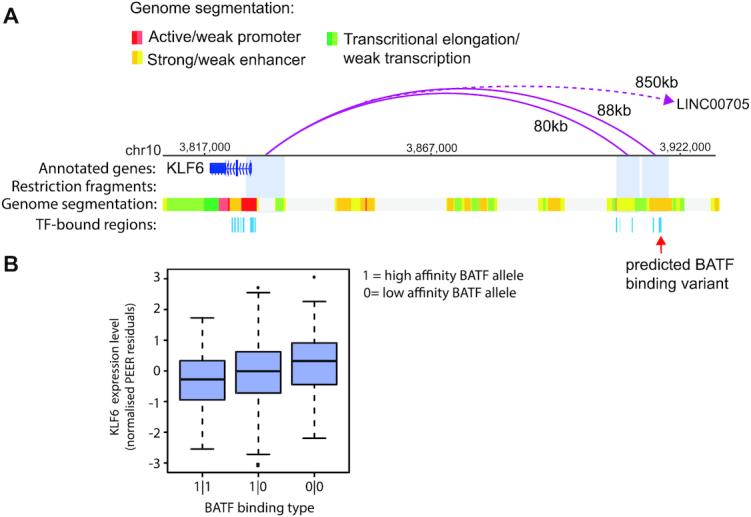
Example of association between a TF binding affinity CRM variant and gene expression. (**A**) Genome browser representation of the distal interactions (pink arches) of *KLF6* promoter in the LCL GM12878, as detected by PCHi-C (49). Two out of the three fragments interacting with *KLF6* promoter are shown; the third fragment, which is located 850 kb away from the *KLF6* promoter and contains the gene *LINC00705*, was omitted due to space constraints. The chromHMM genome segmentation tracks for GM12878 are shown immediately below (80). CRMs at the two distally interacting fragments and the TSS-proximal window are depicted in azure blue. The rightmost CRM, which interacts with the KLF6 promoter 88 kb away, is predicted to impact BATF binding affinity across the 359 LCLs. (**B**) Box plot showing the association between mRNA levels (as measured with RNA-seq by the GEUVADIS consortium) and predicted CRM haplotype with respect to BATF binding affinity in LCLs. *KLF6* expression is significantly associated with BATF binding type (gene-level FDR adjusted *P*-value = 1.21 × 10^−2^, BATF variant *P*-value = 5.16 × 10^−4^, effect size = 0.26).

To identify quantitative associations between TF binding variation at CRMs and the expression of their target genes, we used genome-wide gene expression data from the GEUVADIS project (62) that included 358/359 of the LCLs used in our analysis (with the exception of GM12878). In contrast to traditional eQTL testing, here we devised an approach that prioritizes TF-binding variants and their putative target genes *a priori* and performs testing at the CRM level. In total, we selected 3285 CRMs with predicted variation in the binding for at least one TF (log ratio >0.3). We then tested the association of each CRM haplotype with the expression levels of their target genes defined on the basis of 3D interactions or close spatial proximity (within 9 kb; see ‘Materials and Methods’ section). As evidence of 3D promoter–CRM interactions, we used high-resolution PCHi-C data in GM12878 cells (49,61). The highly reduced search space has enabled testing for associations at the gene level, with all CRMs targeting the same gene and showing TF binding variation included into the regression model (see ‘Materials and Methods’ section). This approach identified 245 ‘eGenes’ with significant associations between predicted TF binding affinity at CRMs and gene expression (16% of 1530 genes tested, at 10% FDR; Supplementary Table S1). In total, 161 ‘proximal’ (within 9 kb) and 101 ‘distal’ TF-CRM affinity variants (with contacts detected by PCHi-C) were found to underlie these associations, corresponding to 26% and 6% of all variants tested, respectively (*t*-test *P*-value <0.05; Supplementary Table S1). Figure 3 shows an example of the detected association between the expression of *KLF6* and variation in the binding affinity of BATF at a distal CRM that is located 88 kb away from *KLF6* promoter and contacts it in 3D according to PCHi-C (gene-level FDR = 1.21 × 10^−2^, BATF variant *P*-value = 5.16 × 10^−4^, effect size = 0.26; the genome segmentation profile shown is based on chromHMM (80)). Individuals homozygous for the high-affinity BATF binding allele showed the lowest levels of *KLF6* expression, while those homozygous for the low-affinity BATF binding alleles showed the highest levels (Figure 3). This suggests that BATF acts as a negative regulator of *KLF6* expression, consistent with its known role as a repressor of AP-1-dependent transcriptional activity (81).

**Figure 4.**
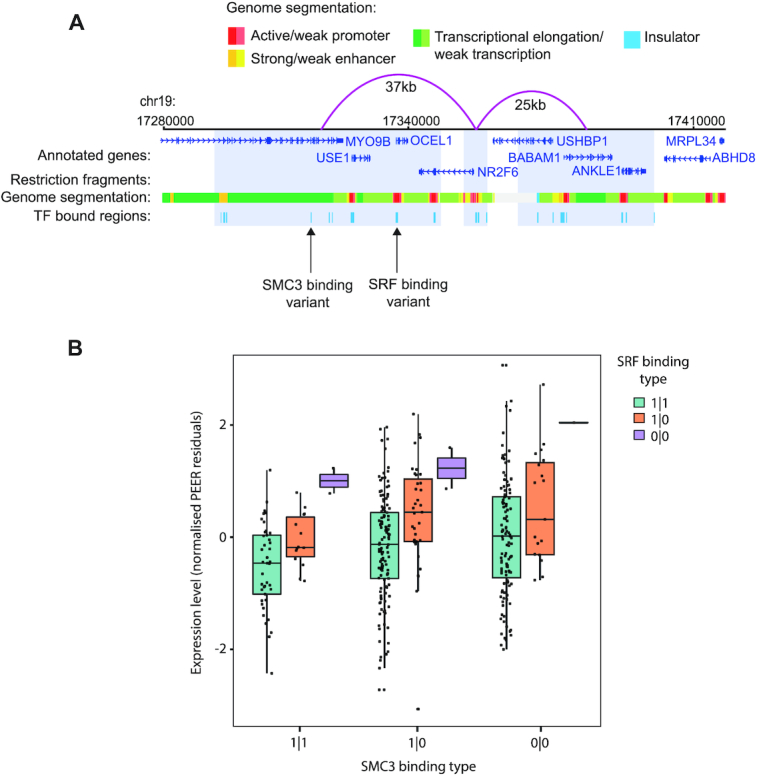
Example of a multivariant association between TF binding affinity at CRMs and their target gene expression. (**A**) Genome browser representation of *NR2F6* promoter distal interactions (represented by pink arches) as detected by PCHi-C (49) in LCL GM12878. The genome segmentation track for GM12878 based on chromHMM (80) is shown immediately below. CRMs at the distally interacting fragments (pale blue) and *NR2F6* TSS-proximal window are depicted in azure blue. The distal fragment downstream of *NR2F6* contains two CRM variants: one 44 kb away from the *NR2F6* promoter and the other 19 kb away, predicted to impact SMC3 and SRF binding affinity, respectively, across the 359 LCLs. (**B**) Association between *NR2F6* mRNA levels and predicted SMC3 and SRF binding affinity haplotypes.

A total of 420/1530 genes (27%) were linked with multiple predicted TF-binding variants (either for different TFs bound at the same CRM or at different CRMs). For 16 of these genes, we detected significant associations between more than one such variant and the expression level. One example is the nuclear receptor gene *NR2F6* whose expression significantly associated with predicted variation in the binding affinities of SMC3 and SRF to distal CRMs located, respectively, 41 and 19 kb away (Figure 4; gene-level FDR = 4.06 × 10^−7^, SMC3 effect size = 0.26, *P*-value = 3 × 10^−4^; SRF effect size = 0.61, *P*-value = 1.19 × 10^−7^).

Owing to the *a priori* prioritization of variants for association testing in our approach (i.e. testing only variants predicted to impact TF binding), we carried out far fewer association tests than in a standard eQTL analysis, thus reducing the multiple testing burden and increasing sensitivity. We therefore asked whether we were able to detect additional associations compared with those reported for a standard eQTL analysis performed by the GEUVADIS project (note that this analysis also used an additional 103 LCLs not included in our study, which were either of non-European ancestry or not genotyped in 1000 Genomes project). To compare our CRM-based association results to GEUVADIS eQTL SNPs, we identified the SNP causing the largest change in affinity for the respective TF at each CRM (192 eQTL SNPs in total at 5% FDR to match the FDR level used by GEUVADIS). Of these, 78 SNPs (42%) were detected as significant by GEUVADIS. Therefore, the remaining 114/192 (58%) eQTL SNPs identified in our approach corresponded to not previously reported associations.

### Threshold-free testing based on TF binding affinities reveals further expression associations

The above-mentioned analysis was performed broadly within the conventional paradigm of eQTL testing, whereby expression was compared across three diploid genotypes (two homozygous and one heterozygous), except that these genotypes corresponded to cases whereby variation was predicted to appreciably disrupt TF binding based on a predefined threshold (we shall refer to this approach as ‘thresholded’), and the gene-CRM combinations were selected for association testing based on PCHi-C data. However, since TF binding affinity haplotypes were defined at the CRM level, more than two haplotypes were commonly observed per CRM with respect to a given TF (in 12–100% cases depending on the TF). In the thresholded approach, we pooled multiple alleles into either ‘high-affinity’ or ‘low-affinity’ haplotypes and disregarded outliers (see ‘Materials and Methods’ section). We reasoned, however, that it is also possible to regress gene expression against normalized TF binding affinities directly without thresholding and haplotype pooling, leading to increased precision and sensitivity of association testing. As expected, this ‘threshold-free’ approach revealed a considerably larger number of genes significantly associated with CRM affinity variants (1033 eGenes at 10% FDR compared with 245 detected in the ‘thresholded’ approach mentioned earlier).

**Figure 5.**
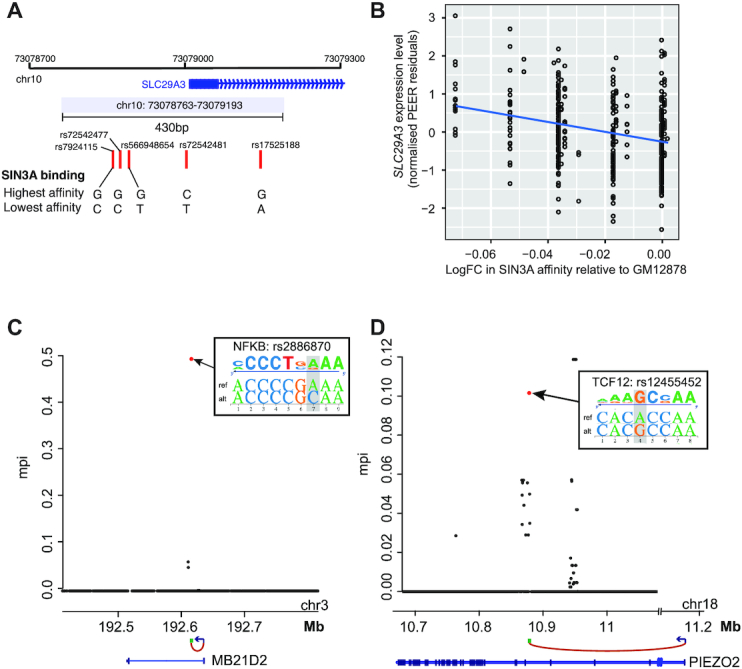
Unthresholded approach for detecting TF binding affinity CRM variant associations with gene expression and their validation using GUESSFM. (**A**) Example of a CRM with multiple SNPs affecting the affinity for the same TF, SIN3A. (**B**) Association between log-fold change in CRM affinity for SIN3A (relative to the highest affinity allele observed in GM12878) and mRNA level (normalized PEER residuals) of the connected gene, *SLC29A3* (gene-level FDR adjusted *P*-value = 1.60 × 10^−4^, *β* = −0.14*)*. (**C**, **D**) Examples of loci, whereby the SNP predicted to have the strongest impact on a CRM’s binding affinity for a given TF has been fine-mapped as a potentially causal variant driving the locus’s association with the expression of a physically connected target gene (GUESSFM ${\rm mppi}\gg 0.001$). (C) eGene: *MB21D2*; eQTL rs2886870, predicted to affect NFKB binding affinity. (D) eGene: *PIEZO2*; eQTL rs12455452, predicted to affect TCF12 binding affinity. See insets for the effects of the SNPs on the respective TF's PWM match.

One challenge arising in the threshold-free approach is that it leads to many more TF affinity CRM variants tested for each gene. Since the same SNPs or those in LD with each other can impact CRM affinity for multiple TFs, the explanatory variables in the regression models are often correlated, posing challenges for the standard ordinary least squares (OLS)-based association testing. Therefore, to detect significant associations in the unthresholded setting, we performed elastic net regression for each of the 895/1033 identified eGenes that were targeted by multiple TF affinity CRM variants. To ascertain the significance of regression coefficients in elastic net regression, we used a covariance test for adaptive linear models (77), identifying 1328 significant CRM–gene associations for the 895 eGenes tested (Supplementary Table S2; see ‘Materials and Methods’ section for details). One example of a newly identified association is between a nucleotide transporter gene *SLC29A3* and the binding affinity of SIN3A at a CRM overlapping with the TSS of *SLC29A3* (gene-level FDR = 1.60 × 10^−4^). Five alternative SIN3A binding affinity haplotypes were observed across the 358 LCLs (Figure 5A), with log-fold changes in affinity for SIN3A (relative to the highest affinity allele of GM12878) ranging from −0.037 to 0.001 (elastic net effect size = −0.14, *P*-value ∼0; Figure 5B). In total, 72% of the TF-CRM variants showing significant associations with gene expression had three or more TF binding affinity haplotypes.

### TF binding affinity variants are highly enriched for causal eQTLs

We asked what proportion of TF-binding variants showing association with target gene expression in our analysis could be fine-mapped as causal purely based on the pattern of association signals in their vicinity, without *a priori* prioritization and pooling of variants per CRM. To this end, we supplied genotype information for ±200 kb windows around the CRMs with detected associations and the respective gene expression data to GUESSFM, a Bayesian fine-mapping approach that accounts for possible multiple causal variants per locus (78). GUESSFM identified at least one causal variant in ∼38% of the analysed CRMs (1807/4718); associations in the remaining CRMs likely could not be fine-mapped due to a lack of statistical power. In ∼30% (548/1807) of CRMs with successful fine mapping, the TF-binding variant showing the strongest association per CRM was ranked as possibly causal (mppi > 0.001), and in the majority of such cases (477/548) this variant was also ranked by GUESSFM among the top five highest scoring variants in the window (see Supplementary Table S3 and Figure 5C and D for examples). In contrast, just 2.6% (48/1807) random variants within the same windows (matched by allele frequency) were detected as potentially causal by GUESSFM, corresponding to a very significant enrichment of fine-mapped variants for those affecting TF binding (Fisher test *P* = 10^−126^).

### Many CRMs associated with distal gene expression show features of epromoters

**Figure 6.**
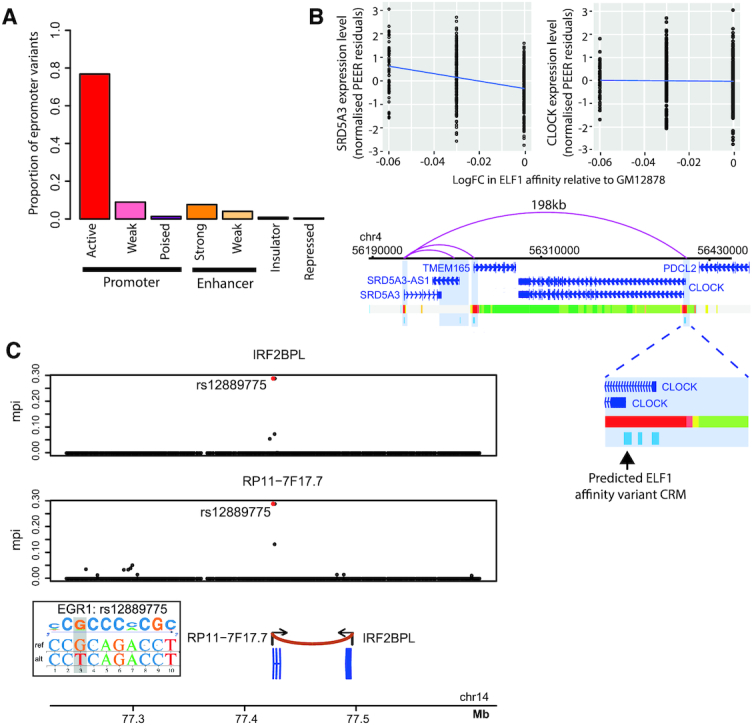
TF binding affinity variants highlight transcriptional regulatory effects of epromoters. (**A**) Bar plot showing the proportion of distal CRMs showing association between TF binding affinity and target gene expression that map in close proximity (within 200 bp) of another gene's TSS overlapping each genome segmentation category (80) for GM12878. (**B**) Top: The association between log FC in CRM affinity for ELF1 relative to the highest affinity allele of GM12878 and mRNA level (normalized PEER residuals) of *SRD5A3* and *CLOCK*. Bottom: Genome browser representation of the distal interactions detected by PCHi-C (49) for SRD5A3, with CRMs identified at each fragment as well as the proximal window depicted in light blue. The genome segmentation track for GM12878 based on chromHMM (80) is shown immediately below (see Figure 4A for the colour key). Inset: Enlarged view of an interacting fragment containing three CRMs, one of which harbours variants predicted to impact ELF1 binding affinity and overlaps with the *CLOCK* promoter. (**C**) Colocalization analysis showing shared association between epromoter-located SNP rs12889775 and the expression of both its distal and proximal genes (*IRF2BPL*, top, and lncRNA *RP11-7F17.7*, bottom, respectively). Posterior probability of shared association estimated by the coloc software *P*_H4_ = 0.997. This SNP is predicted to affect the epromoter’s binding affinity for EGR1 (see inset).

We noted that a large number of distal CRMs showing association between TF binding affinity and target gene expression (224 CRMs, 243 TF-CRM variants; Supplementary Table S4) and connecting to the distal gene promoters in 3D based on PCHi-C also mapped in close proximity (within 200 bp) of the TSS of either one or more other genes (165 and 59 CRMs, respectively, and 284 eGenes; note that the number of eGenes is greater than that of CRMs due to some CRMs mapping in close proximity of multiple TSSs). The absolute majority (87%) of these CRMs localized within chromatin segments with the characteristic features of gene promoters (Figure 6A). Taken together, this suggested that some promoter regions might act as distal regulatory regions of other genes, whose promoters they physically contact. This class of CRMs with dual promoter and enhancer activity were independently identified in two recent studies (63,64). We shall follow Dao *et al.* (63) in referring to these CRMs as ‘epromoters’.

Most genes located in the immediate vicinity of the identified epromoters were appreciably expressed in LCLs (232/284, 82%). However, TF binding variation at nearly two-thirds of epromoters whose proximal gene was expressed (139 variants, 64.7%; see Supplementary Table S2) showed detectable association with a distal gene alone in independent tests (assessed with the threshold-free approach). For example, variation in ELF1 binding affinity at a CRM that shows promoter-associated chromatin marks and localizes within 200 bp from the TSS of *CLOCK* gene does not affect *CLOCK* expression. Instead, it associates with expression of *SRD5A3* located 198 kb away, whose promoter it contacts in 3D as detected by PCHi-C (Figure 6B; *SRD5A3*: gene-level FDR = 3.33 × 10^−21^, ELF1 elastic net *P*-value = 0, ELF1 elastic net *β* = −0.21; *CLOCK*: gene-level FDR = 0.88).

The remaining 76 TF–epromoter CRM variants showed associations between with the expression levels of both distal and proximal genes. To obtain formal evidence that these associations were indeed driven by the same variant and not by different variants in LD with each other, we used colocalization analysis (79), while accounting for multiple independent associations (see ‘Materials and Methods’ section). We submitted to this analysis the most tractable subset of seven epromoters, for which the association of the respective TF-binding variant with distal gene expression was independently confirmed by fine mapping (GUESSFM mppi > 0.001). At 6/7 analysed epromoters, we found prevailing evidence of shared association signals for both the proximal and distal genes (*P*_H4_ > 0.66; Supplementary Table S5). An example of such high-confidence shared signal is variation in EGR1 binding affinity at the epromoter of lncRNA *RP11-71F7.7* that associates with the expression of both *RP11-71F7.7* and another gene, *IRF2BPL* (Figure 6C). The promoters of these two genes, transcribed in a convergent orientation, are ∼69 kb apart and contact each other in 3D as detected by PCHi-C.

Taken together, our findings confirm long-range transcriptional regulation by epromoters and suggest that regulatory variants within these elements may have both shared and independent effects on the expression of their proximal and distal target genes.

## DISCUSSION

In this study, we have generated an atlas of CRM variants predicted to affect TF binding in LCLs and established their associations with the expression of their putative target genes. The key methodological innovations of our work are the prioritization and pooling of variants at CRM level using a biophysical model of TF binding affinity, as well as the prioritization of CRM target genes based on high-resolution PCHi-C data. We perform variant and target gene prioritization *a priori* of eQTL testing to increase detection sensitivity and the likelihood of revealing causal associations. Using this strategy, we have detected ∼1300 associations between CRM variants and target gene expression in LCLs. Our approach reveals eQTLs detected at high sensitivity, whose enrichment for causal variants is validated by statistical fine-mapping analysis and by comparison with independently generated MPRA data. Notably, we find that many TF-binding variants showing associations with distal gene expression localize to the promoters of other genes, in support of the recently characterized class of ‘epromoter’ regulatory elements (63,64).

The atlas of binding variants generated in this study is based on EUR individuals from 1000 Genomes Project release and extends our earlier work using the pilot data from the same project (32). Importantly, unlike in our earlier work (32) and other published resources (82,83), here we have used a biophysical model (39) that aggregates TF binding affinities across the whole CRM to increase sensitivity. This model has been used successfully in previous studies of cis-regulatory control (84–87). The relevance of integrating information at CRM level is further highlighted by recent studies showing the importance of weak TF binding events in gene regulation (42,88,89). Therefore, our approach provides a biologically meaningful paradigm for variant pooling at CRM level.

In choosing to quantify variant effects on TF binding in terms of affinity changes, we were attracted by the direct biological interpretability of this metric. A complementary strategy to score TF affinity at CRM level is provided by hidden Markov models (HMMs) (90–92). HMM-based frameworks can be useful, for example, for modelling effects of TF cooperativity (90,91), which could be incorporated into future variant prioritization frameworks. Machine learning algorithms, and particularly deep neural networks, may potentially model even more complex relationships between DNA sequence and TF binding (68,93–95), although typically at the expense of direct biological interpretability. Reassuringly, our predicted variant effects on TF binding affinity are generally correlated with the predictions of the well-established deep-learning model DeepSea (68). Notably, the biophysical model used in our study constitutes one of the layers in a recently proposed fully interpretable deep learning model of *Drosophila* transcriptional control (96), highlighting the continued relevance of this approach.

Predicting the effects of genetic variants on the expression of distal genes is a highly challenging task. To our knowledge, no machine learning model currently generates such predictions for CRM–promoter interaction distances beyond ∼50 kb, reinforcing the importance of evidence from functional genomics, chromosomal conformation and population genetics studies for understanding long-range variant effects. Here, to prioritize the target genes of distal regulatory variants at high sensitivity and resolution, we have taken advantage of PCHi-C data. PCHi-C provides a 15–20-fold enrichment of promoter interactions over the conventional Hi-C technology (48–50) that was previously used in variant effect analyses (95,97). Theoretically, the effects of nucleotide variants on TF binding can also be incorporated as a prior in global association analyses such as fgwas (98), and have already been used in eQTL fine mapping (99). A formal eQTL testing framework using 3D interaction data as a prior is, however, yet to be established.

Our finding that polymorphic TF binding sites at distal CRMs show gene expression associations less frequently compared with proximal regions is consistent with the high degree of redundancy of long-range regulatory elements (5–7,100,101). Predicting the extent of buffering of regulatory variation for a given CRM with a reasonable precision is an important problem that is currently highly challenging due to the sheer number of parameters and the relatively small sample sizes of multi-individual expression datasets. Profiling gene expression in the emerging much larger genotype panels such as UK10K (102) and UK Biobank (103) may provide opportunities for addressing this question.

We observe that a large proportion of CRMs showing associations with the expression of physically connected distal genes are located in the promoter regions of other genes. This finding provides support to the recently characterized class of ‘epromoters’: elements with a dual proximal and distal activity that were discovered on the large scale using high-throughput reporter and CRISPR knockout screens (63–65). Empirically, chromosomal interactions between epromoter CRMs and their distal targets fall into the category of promoter–promoter interactions. Until recently, these interactions have been considered primarily in the context of coordinated gene activation or repression (104–106), such as that observed in *Hox* and histone clusters (104,107). That some promoter–promoter contacts reflect relationships between epromoters and their distal target genes suggests that these contacts may show functionally and possibly even structurally distinct properties.

We show that TF binding variation at epromoters may or may not co-associate with the expression of both proximal and distal genes at the same time. Shared association is consistent with the findings from massively parallel reporter assays that the same sequences are often involved in mediating both promoter and enhancer activity *in vitro* (108). It is possible that some non-shared effects observed in our study *in vivo* are underpinned by the role of the affected TFs in mediating long-range contacts. Additionally, epromoter elements may show different degrees of redundancy with respect to the proximal and distal target genes.

Overall, our analysis demonstrates the potential of model-based prioritization and pooling of variants *a priori* of testing for increasing the sensitivity of identifying individual associations and revealing their shared biological properties.

## DATA AVAILABILITY

The list of the detected TF affinity CRM variants, the full data on CRM variant–gene expression associations and the raw output of GUESSFM fine mapping have been uploaded to OSF (https://osf.io/fa4u7/). The scripts used to generate TF binding affinity variants and perform expression association testing have been uploaded to the same OSF repository. Scripts used for running GUESSFM and coloc are available from https://github.com/chr1swallace/eqtlfm-mikhail/.

## Supplementary Material

gkaa123_Supplemental_Files

## Notes

Present address: Joanna Mitchelmore, Novartis Institutes of Biomedical Research, Novartis Campus, Fabrikstrasse 16, 4056 Basel, Switzerland.

## References

[1] Suryamohan K., Halfon M.S. Identifying transcriptional cis-regulatory modules in animal genomes. Wiley Interdiscip. Rev. Dev. Biol. 2015; 4:59–84. 25704908 10.1002/wdev.168 PMC4339228

[2] Whitaker J.W., Nguyen T.T., Zhu Y., Wildberg A., Wang W. Computational schemes for the prediction and annotation of enhancers from epigenomic assays. Methods. 2015; 72:86–94. 25461775 10.1016/j.ymeth.2014.10.008 PMC4778972

[3] Mei S., Qin Q., Wu Q., Sun H., Zheng R., Zang C., Zhu M., Wu J., Shi X., Taing L. et al. Cistrome Data Browser: a data portal for ChIP-Seq and chromatin accessibility data in human and mouse. Nucleic Acids Res. 2017; 45:D658–D662. 27789702 10.1093/nar/gkw983 PMC5210658

[4] The ENCODE Project Consortium An integrated encyclopedia of DNA elements in the human genome. Nature. 2012; 489:57–74. 22955616 10.1038/nature11247 PMC3439153

[5] Spivakov M. Spurious transcription factor binding: non-functional or genetically redundant?. Bioessays. 2014; 36:798–806. 24888900 10.1002/bies.201400036 PMC4230394

[6] Osterwalder M., Barozzi I., Tissières V., Fukuda-Yuzawa Y., Mannion B.J., Afzal S.Y., Lee E.A., Zhu Y., Plajzer-Frick I., Pickle C.S. et al. Enhancer redundancy provides phenotypic robustness in mammalian development. Nature. 2018; 554:239–243. 29420474 10.1038/nature25461 PMC5808607

[7] Frankel N., Davis G.K., Vargas D., Wang S., Payre F., Stern D.L. Phenotypic robustness conferred by apparently redundant transcriptional enhancers. Nature. 2010; 466:490–493. 20512118 10.1038/nature09158 PMC2909378

[8] Diao Y., Li B., Meng Z., Jung I., Lee A.Y., Dixon J., Maliskova L., Guan K.-L., Shen Y., Ren B. A new class of temporarily phenotypic enhancers identified by CRISPR/Cas9-mediated genetic screening. Genome Res. 2016; 26:397–405. 26813977 10.1101/gr.197152.115 PMC4772021

[9] Fulco C.P., Munschauer M., Anyoha R., Munson G., Grossman S.R., Perez E.M., Kane M., Cleary B., Lander E.S., Engreitz J.M. Systematic mapping of functional enhancer–promoter connections with CRISPR interference. Science. 2016; 354:769–773. 27708057 10.1126/science.aag2445 PMC5438575

[10] Yao L., Berman B.P., Farnham P.J. Demystifying the secret mission of enhancers: linking distal regulatory elements to target genes. Crit. Rev. Biochem. Mol. Biol. 2015; 50:550–573. 26446758 10.3109/10409238.2015.1087961 PMC4666684

[11] Gilad Y., Rifkin S.A., Pritchard J.K. Revealing the architecture of gene regulation: the promise of eQTL studies. Trends Genet. 2008; 24:408–415. 18597885 10.1016/j.tig.2008.06.001 PMC2583071

[12] Majewski J., Pastinen T. The study of eQTL variations by RNA-seq: from SNPs to phenotypes. Trends Genet. 2011; 27:72–79. 21122937 10.1016/j.tig.2010.10.006

[13] Stranger B.E., Raj T. Genetics of human gene expression. Curr. Opin. Genet. Dev. 2013; 23:627–634. 24238872 10.1016/j.gde.2013.10.004

[14] Lappalainen T. Functional genomics bridges the gap between quantitative genetics and molecular biology. Genome Res. 2015; 25:1427–1431. 26430152 10.1101/gr.190983.115 PMC4579327

[15] Tian Lu, Tian L., Quitadamo A., Lin F., Shi X. Methods for population-based eQTL analysis in human genetics. Tsinghua Sci. Technol. 2014; 19:624–634.

[16] Battle A., Montgomery S.B. Determining causality and consequence of expression quantitative trait loci. Hum. Genet. 2014; 133:727–735. 24770875 10.1007/s00439-014-1446-0 PMC4077614

[17] Yashiro-Ohtani Y., Wang H., Zang C., Arnett K.L., Bailis W., Ho Y., Knoechel B., Lanauze C., Louis L., Forsyth K.S. et al. Long-range enhancer activity determines Myc sensitivity to Notch inhibitors in T cell leukemia. Proc. Natl. Acad. Sci. U.S.A. 2014; 111:E4946–E4953. 25369933 10.1073/pnas.1407079111 PMC4246292

[18] Lettice L.A., Heaney S.J.H., Purdie L.A., Li L., de Beer P., Oostra B.A., Goode D., Elgar G., Hill R.E., de Graaff E. A long-range Shh enhancer regulates expression in the developing limb and fin and is associated with preaxial polydactyly. Hum. Mol. Genet. 2003; 12:1725–1735. 12837695 10.1093/hmg/ddg180

[19] Zhou H.Y., Katsman Y., Dhaliwal N.K., Davidson S., Macpherson N.N., Sakthidevi M., Collura F., Mitchell J.A. A Sox2 distal enhancer cluster regulates embryonic stem cell differentiation potential. Genes Dev. 2014; 28:2699–2711. 25512558 10.1101/gad.248526.114 PMC4265674

[20] Corradin O., Saiakhova A., Akhtar-Zaidi B., Myeroff L., Willis J., Cowper-Sal lari R., Lupien M., Markowitz S., Scacheri P.C. Combinatorial effects of multiple enhancer variants in linkage disequilibrium dictate levels of gene expression to confer susceptibility to common traits. Genome Res. 2014; 24:1–13. 24196873 10.1101/gr.164079.113 PMC3875850

[21] Spitz F., Furlong E.E.M. Transcription factors: from enhancer binding to developmental control. Nat. Rev. Genet. 2012; 13:613–626. 22868264 10.1038/nrg3207

[22] Long H.K., Prescott S.L., Wysocka J. Ever-changing landscapes: transcriptional enhancers in development and evolution. Cell. 2016; 167:1170–1187. 27863239 10.1016/j.cell.2016.09.018 PMC5123704

[23] Gonen N., Futtner C.R., Wood S., Garcia-Moreno S.A., Salamone I.M., Samson S.C., Sekido R., Poulat F., Maatouk D.M., Lovell-Badge R. Sex reversal following deletion of a single distal enhancer of *Sox9*. Science. 2018; 360:1469–1473. 29903884 10.1126/science.aas9408 PMC6034650

[24] Miguel-Escalada I., Pasquali L., Ferrer J. Transcriptional enhancers: functional insights and role in human disease. Curr. Opin. Genet. Dev. 2015; 33:71–76. 26433090 10.1016/j.gde.2015.08.009 PMC4720706

[25] Gallone G., Haerty W., Disanto G., Ramagopalan S.V., Ponting C.P., Berlanga-Taylor A.J. Identification of genetic variants affecting vitamin D receptor binding and associations with autoimmune disease. Hum. Mol. Genet. 2017; 26:2164–2176. 28335003 10.1093/hmg/ddx092 PMC5886188

[26] Ding Z., Ni Y., Timmer S.W., Lee B.-K., Battenhouse A., Louzada S., Yang F., Dunham I., Crawford G.E., Lieb J.D. et al. Quantitative genetics of CTCF binding reveal local sequence effects and different modes of X-chromosome association. PLoS Genet. 2014; 10:e1004798. 25411781 10.1371/journal.pgen.1004798 PMC4238955

[27] Kasowski M., Kyriazopoulou-Panagiotopoulou S., Grubert F., Zaugg J.B., Kundaje A., Liu Y., Boyle A.P., Zhang Q.C., Zakharia F., Spacek D.V. et al. Extensive variation in chromatin states across humans. Science. 2013; 342:750–752. 24136358 10.1126/science.1242510 PMC4075767

[28] Kilpinen H., Waszak S.M., Gschwind A.R., Raghav S.K., Witwicki R.M., Orioli A., Migliavacca E., Wiederkehr M., Gutierrez-Arcelus M., Panousis N.I. et al. Coordinated effects of sequence variation on DNA binding, chromatin structure, and transcription. Science. 2013; 342:744–747. 24136355 10.1126/science.1242463 PMC5502466

[29] Maurano M.T., Wang H., Kutyavin T., Stamatoyannopoulos J.A. Widespread site-dependent buffering of human regulatory polymorphism. PLoS Genet. 2012; 8:e1002599. 22457641 10.1371/journal.pgen.1002599 PMC3310774

[30] Kasowski M., Grubert F., Heffelfinger C., Hariharan M., Asabere A., Waszak S.M., Habegger L., Rozowsky J., Shi M., Urban A.E. et al. Variation in transcription factor binding among humans. Science. 2010; 328:232–235. 20299548 10.1126/science.1183621 PMC2938768

[31] Stormo G.D. DNA binding sites: representation and discovery. Bioinformatics. 2000; 16:16–23. 10812473 10.1093/bioinformatics/16.1.16

[32] Spivakov M., Akhtar J., Kheradpour P., Beal K., Girardot C., Koscielny G., Herrero J., Kellis M., Furlong E.E.M., Birney E. Analysis of variation at transcription factor binding sites in *Drosophila* and humans. Genome Biol. 2012; 13:R49. 22950968 10.1186/gb-2012-13-9-r49 PMC3491393

[33] Kim J., He X., Sinha S. Evolution of regulatory sequences in 12 *Drosophila* species. PLoS Genet. 2009; 5:e1000330. 19132088 10.1371/journal.pgen.1000330 PMC2607023

[34] Chen K., van Nimwegen E., Rajewsky N., Siegal M.L. Correlating gene expression variation with cis-regulatory polymorphism in *Saccharomyces cerevisiae*. Genome Biol. Evol. 2010; 2:697–707. 20829281 10.1093/gbe/evq054 PMC2953268

[35] Hertz G.Z., Stormo G.D. Identifying DNA and protein patterns with statistically significant alignments of multiple sequences. Bioinformatics. 1999; 15:563–577. 10487864 10.1093/bioinformatics/15.7.563

[36] Grant C.E., Bailey T.L., Noble W.S. FIMO: scanning for occurrences of a given motif. Bioinformatics. 2011; 27:1017–1018. 21330290 10.1093/bioinformatics/btr064 PMC3065696

[37] Berg O.G., von Hippel P.H. Selection of DNA binding sites by regulatory proteins. Statistical–mechanical theory and application to operators and promoters. J. Mol. Biol. 1987; 193:723–750. 3612791 10.1016/0022-2836(87)90354-8

[38] Ruan S., Stormo G.D. Inherent limitations of probabilistic models for protein–DNA binding specificity. PLoS Comput. Biol. 2017; 13:e1005638. 28686588 10.1371/journal.pcbi.1005638 PMC5521849

[39] Roider H.G., Kanhere A., Manke T., Vingron M. Predicting transcription factor affinities to DNA from a biophysical model. Bioinformatics. 2007; 23:134–141. 17098775 10.1093/bioinformatics/btl565

[40] Manke T., Roider H.G., Vingron M. Statistical modeling of transcription factor binding affinities predicts regulatory interactions. PLoS Comput. Biol. 2008; 4:e1000039. 18369429 10.1371/journal.pcbi.1000039 PMC2266803

[41] Ramos A.I., Barolo S. Low-affinity transcription factor binding sites shape morphogen responses and enhancer evolution. Philos. Trans. R. Soc. Lond. B: Biol. Sci. 2013; 368:20130018. 24218631 10.1098/rstb.2013.0018 PMC3826492

[42] Farley E.K., Olson K.M., Zhang W., Brandt A.J., Rokhsar D.S., Levine M.S. Suboptimization of developmental enhancers. Science. 2015; 350:325–328. 26472909 10.1126/science.aac6948 PMC4970741

[43] He X., Duque T.S.P.C., Sinha S. Evolutionary origins of transcription factor binding site clusters. Mol. Biol. Evol. 2012; 29:1059–1070. 22075113 10.1093/molbev/msr277 PMC3278477

[44] Krivega I., Dean A. Enhancer and promoter interactions—long distance calls. Curr. Opin. Genet. Dev. 2012; 22:79–85. 22169023 10.1016/j.gde.2011.11.001 PMC3342482

[45] Ong C.-T., Corces V.G. Enhancer function: new insights into the regulation of tissue-specific gene expression. Nat. Rev. Genet. 2011; 12:283–293. 21358745 10.1038/nrg2957 PMC3175006

[46] Maeso I., Acemel R.D., Gómez-Skarmeta J.L. Cis-regulatory landscapes in development and evolution. Curr. Opin. Genet. Dev. 2017; 43:17–22. 27842294 10.1016/j.gde.2016.10.004

[47] Schmitt A.D., Hu M., Ren B. Genome-wide mapping and analysis of chromosome architecture. Nat. Rev. Mol. Cell Biol. 2016; 17:743–755. 27580841 10.1038/nrm.2016.104 PMC5763923

[48] Schoenfelder S., Furlan-Magaril M., Mifsud B., Tavares-Cadete F., Sugar R., Javierre B.-M., Nagano T., Katsman Y., Sakthidevi M., Wingett S.W. et al. The pluripotent regulatory circuitry connecting promoters to their long-range interacting elements. Genome Res. 2015; 25:582–597. 25752748 10.1101/gr.185272.114 PMC4381529

[49] Mifsud B., Tavares-Cadete F., Young A.N., Sugar R., Schoenfelder S., Ferreira L., Wingett S.W., Andrews S., Grey W., Ewels P.A. et al. Mapping long-range promoter contacts in human cells with high-resolution Capture Hi-C. Nat. Genet. 2015; 47:598–606. 25938943 10.1038/ng.3286

[50] Sahlén P., Abdullayev I., Ramsköld D., Matskova L., Rilakovic N., Lötstedt B., Albert T.J., Lundeberg J., Sandberg R. Genome-wide mapping of promoter-anchored interactions with close to single-enhancer resolution. Genome Biol. 2015; 16:156. 26313521 10.1186/s13059-015-0727-9 PMC4557751

[51] Javierre B.M., Burren O.S., Wilder S.P., Kreuzhuber R., Hill S.M., Sewitz S., Cairns J., Wingett S.W., Várnai C., Thiecke M.J. et al. Lineage-specific genome architecture links enhancers and non-coding disease variants to target gene promoters. Cell. 2016; 167:1369–1384. 27863249 10.1016/j.cell.2016.09.037 PMC5123897

[52] Choy M.-K., Javierre B.M., Williams S.G., Baross S.L., Liu Y., Wingett S.W., Akbarov A., Wallace C., Freire-Pritchett P., Rugg-Gunn P.J. et al. Promoter interactome of human embryonic stem cell-derived cardiomyocytes connects GWAS regions to cardiac gene networks. Nat. Commun. 2018; 9:2526. 29955040 10.1038/s41467-018-04931-0 PMC6023870

[53] Burren O.S., Rubio García A., Javierre B.-M., Rainbow D.B., Cairns J., Cooper N.J., Lambourne J.J., Schofield E., Castro Dopico X., Ferreira R.C. et al. Chromosome contacts in activated T cells identify autoimmune disease candidate genes. Genome Biol. 2017; 18:165. 28870212 10.1186/s13059-017-1285-0 PMC5584004

[54] Petersen R., Lambourne J.J., Javierre B.M., Grassi L., Kreuzhuber R., Ruklisa D., Rosa I.M., Tomé A.R., Elding H., van Geffen J.P. et al. Platelet function is modified by common sequence variation in megakaryocyte super enhancers. Nat. Commun. 2017; 8:16058. 28703137 10.1038/ncomms16058 PMC5511350

[55] Jäger R., Migliorini G., Henrion M., Kandaswamy R., Speedy H.E., Heindl A., Whiffin N., Carnicer M.J., Broome L., Dryden N. et al. Capture Hi-C identifies the chromatin interactome of colorectal cancer risk loci. Nat. Commun. 2015; 6:6178. 25695508 10.1038/ncomms7178 PMC4346635

[56] Dryden N.H., Broome L.R., Dudbridge F., Johnson N., Orr N., Schoenfelder S., Nagano T., Andrews S., Wingett S., Kozarewa I. et al. Unbiased analysis of potential targets of breast cancer susceptibility loci by Capture Hi-C. Genome Res. 2014; 24:1854–1868. 25122612 10.1101/gr.175034.114 PMC4216926

[57] McGovern A., Schoenfelder S., Martin P., Massey J., Duffus K., Plant D., Yarwood A., Pratt A.G., Anderson A.E., Isaacs J.D. et al. Capture Hi-C identifies a novel causal gene, IL20RA, in the pan-autoimmune genetic susceptibility region 6q23. Genome Biol. 2016; 17:212. 27799070 10.1186/s13059-016-1078-x PMC5088679

[58] Martin P., McGovern A., Massey J., Schoenfelder S., Duffus K., Yarwood A., Barton A., Worthington J., Fraser P., Eyre S. et al. Identifying causal genes at the multiple sclerosis associated region 6q23 using Capture Hi-C. PLoS One. 2016; 11:e0166923. 27861577 10.1371/journal.pone.0166923 PMC5115837

[59] Baxter J.S., Leavy O.C., Dryden N.H., Maguire S., Johnson N., Fedele V., Simigdala N., Martin L.-A., Andrews S., Wingett S.W. et al. Capture Hi-C identifies putative target genes at 33 breast cancer risk loci. Nat. Commun. 2018; 9:1028. 29531215 10.1038/s41467-018-03411-9 PMC5847529

[60] The 1000 Genomes Project Consortium An integrated map of genetic variation from 1,092 human genomes. Nature. 2012; 491:56–65. 23128226 10.1038/nature11632 PMC3498066

[61] Cairns J., Freire-Pritchett P., Wingett S.W., Várnai C., Dimond A., Plagnol V., Zerbino D., Schoenfelder S., Javierre B.-M., Osborne C. et al. CHiCAGO: robust detection of DNA looping interactions in Capture Hi-C data. Genome Biol. 2016; 17:127. 27306882 10.1186/s13059-016-0992-2 PMC4908757

[62] Lappalainen T., Sammeth M., Friedländer M.R., ’t Hoen P.A.C., Monlong J., Rivas M.A., Gonzàlez-Porta M., Kurbatova N., Griebel T., Ferreira P.G. et al. Transcriptome and genome sequencing uncovers functional variation in humans. Nature. 2013; 501:506–511. 24037378 10.1038/nature12531 PMC3918453

[63] Dao L.T.M., Galindo-Albarrán A.O., Castro-Mondragon J.A., Andrieu-Soler C., Medina-Rivera A., Souaid C., Charbonnier G., Griffon A., Vanhille L., Stephen T. et al. Genome-wide characterization of mammalian promoters with distal enhancer functions. Nat. Genet. 2017; 49:1073–1081. 28581502 10.1038/ng.3884

[64] Diao Y., Fang R., Li B., Meng Z., Yu J., Qiu Y., Lin K.C., Huang H., Liu T., Marina R.J. et al. A tiling-deletion-based genetic screen for cis-regulatory element identification in mammalian cells. Nat. Methods. 2017; 14:629–635. 28417999 10.1038/nmeth.4264 PMC5490986

[65] Dao L.T.M., Spicuglia S. Transcriptional regulation by promoters with enhancer function. Transcription. 2018; 9:307–314. 29889606 10.1080/21541264.2018.1486150 PMC6150634

[66] Kheradpour P., Kellis M. Systematic discovery and characterization of regulatory motifs in ENCODE TF binding experiments. Nucleic Acids Res. 2014; 42:2976–2987. 24335146 10.1093/nar/gkt1249 PMC3950668

[67] Zuo C., Shin S., Keleş S. atSNP: transcription factor binding affinity testing for regulatory SNP detection. Bioinformatics. 2015; 31:3353–3355. 26092860 10.1093/bioinformatics/btv328 PMC4643619

[68] Zhou J., Troyanskaya O.G. Predicting effects of noncoding variants with deep learning-based sequence model. Nat. Methods. 2015; 12:931–934. 26301843 10.1038/nmeth.3547 PMC4768299

[69] Degner J.F., Pai A.A., Pique-Regi R., Veyrieras J.-B., Gaffney D.J., Pickrell J.K., De Leon S., Michelini K., Lewellen N., Crawford G.E. et al. DNase I sensitivity QTLs are a major determinant of human expression variation. Nature. 2012; 482:390–394. 22307276 10.1038/nature10808 PMC3501342

[70] Hinrichs A.S. The UCSC Genome Browser Database: update 2006. Nucleic Acids Res. 2006; 34:D590–D598. 16381938 10.1093/nar/gkj144 PMC1347506

[71] Tehranchi A., Hie B., Dacre M., Kaplow I., Pettie K., Combs P., Fraser H.B. Fine-mapping-regulatory variants in diverse human populations. Elife. 2019; 8,:e39595. 30650056 10.7554/eLife.39595 PMC6335058

[72] Tewhey R., Kotliar D., Park D.S., Liu B., Winnicki S., Reilly S.K., Andersen K.G., Mikkelsen T.S., Lander E.S., Schaffner S.F. et al. Direct identification of hundreds of expression-modulating variants using a multiplexed reporter assay. Cell. 2018; 172:1132–1134. 29474912 10.1016/j.cell.2018.02.021

[73] Durinck S., Spellman P.T., Birney E., Huber W. Mapping identifiers for the integration of genomic datasets with the R/Bioconductor package biomaRt. Nat. Protoc. 2009; 4:1184–1191. 19617889 10.1038/nprot.2009.97 PMC3159387

[74] Stegle O., Parts L., Durbin R., Winn J. A Bayesian framework to account for complex non-genetic factors in gene expression levels greatly increases power in eQTL studies. PLoS Comput. Biol. 2010; 6:e1000770. 20463871 10.1371/journal.pcbi.1000770 PMC2865505

[75] Kolesnikov N., Hastings E., Keays M., Melnichuk O., Tang Y.A., Williams E., Dylag M., Kurbatova N., Brandizi M., Burdett T. et al. ArrayExpress update—simplifying data submissions. Nucleic Acids Res. 2015; 43:D1113–D1116. 25361974 10.1093/nar/gku1057 PMC4383899

[76] Aulchenko Y.S., Ripke S., Isaacs A., van Duijn C.M. GenABEL: an R library for genome-wide association analysis. Bioinformatics. 2007; 23:1294–1296. 17384015 10.1093/bioinformatics/btm108

[77] Lockhart R., Taylor J., Tibshirani R.J., Tibshirani R. A significance test for the lasso. Ann. Stat. 2014; 42:413–468. 25574062 10.1214/13-AOS1175 PMC4285373

[78] Wallace C., Cutler A.J., Pontikos N., Pekalski M.L., Burren O.S., Cooper J.D., García A.R., Ferreira R.C., Guo H., Walker N.M. et al. Dissection of a complex disease susceptibility region using a Bayesian stochastic search approach to fine mapping. PLoS Genet. 2015; 11:e1005272. 26106896 10.1371/journal.pgen.1005272 PMC4481316

[79] Giambartolomei C., Vukcevic D., Schadt E.E., Franke L., Hingorani A.D., Wallace C., Plagnol V. Bayesian test for colocalisation between pairs of genetic association studies using summary statistics. PLoS Genet. 2014; 10:e1004383. 24830394 10.1371/journal.pgen.1004383 PMC4022491

[80] Ernst J., Kellis M. Chromatin-state discovery and genome annotation with ChromHMM. Nat. Protoc. 2017; 12:2478–2492. 29120462 10.1038/nprot.2017.124 PMC5945550

[81] Williams K.L., Nanda I., Lyons G.E., Kuo C.T., Schmid M., Leiden J.M., Kaplan M.H., Taparowsky E.J. Characterization of murine BATF: a negative regulator of activator protein-1 activity in the thymus. Eur. J. Immunol. 2001; 31:1620–1627. 11466704 10.1002/1521-4141(200105)31:5<1620::aid-immu1620>3.0.co;2-3

[82] Ward L.D., Kellis M. HaploReg v4: systematic mining of putative causal variants, cell types, regulators and target genes for human complex traits and disease. Nucleic Acids Res. 2016; 44:D877–D881. 26657631 10.1093/nar/gkv1340 PMC4702929

[83] Kumar S., Ambrosini G., Bucher P. SNP2TFBS—a database of regulatory SNPs affecting predicted transcription factor binding site affinity. Nucleic Acids Res. 2017; 45:D139–D144. 27899579 10.1093/nar/gkw1064 PMC5210548

[84] Roider H.G., Manke T., O’Keeffe S., Vingron M., Haas S.A. PASTAA: identifying transcription factors associated with sets of co-regulated genes. Bioinformatics. 2009; 25:435–442. 19073590 10.1093/bioinformatics/btn627 PMC2642637

[85] Schmidt F., Gasparoni N., Gasparoni G., Gianmoena K., Cadenas C., Polansky J.K., Ebert P., Nordström K., Barann M., Sinha A. et al. Combining transcription factor binding affinities with open-chromatin data for accurate gene expression prediction. Nucleic Acids Res. 2017; 45:54–66. 27899623 10.1093/nar/gkw1061 PMC5224477

[86] Costa I.G., Roider H.G., do Rego T.G., de Carvalho F., de A.T. Predicting gene expression in T cell differentiation from histone modifications and transcription factor binding affinities by linear mixture models. BMC Bioinformatics. 2011; 12(Suppl. 1):S29. 21342559 10.1186/1471-2105-12-S1-S29 PMC3044284

[87] Junion G., Spivakov M., Girardot C., Braun M., Gustafson E.H., Birney E., Furlong E.E.M. A transcription factor collective defines cardiac cell fate and reflects lineage history. Cell. 2012; 148:473–486. 22304916 10.1016/j.cell.2012.01.030

[88] de Boer C.G., Vaishnav E.D., Sadeh R., Abeyta E.L., Friedman N., Regev A. Deciphering eukaryotic gene-regulatory logic with 100 million random promoters. Nat. Biotechnol. 2019; 38:56–65. 31792407 10.1038/s41587-019-0315-8 PMC6954276

[89] Bruno L., Ramlall V., Studer R.A., Sauer S., Bradley D., Dharmalingam G., Carroll T., Ghoneim M., Chopin M., Nutt S.L. et al. Selective deployment of transcription factor paralogs with submaximal strength facilitates gene regulation in the immune system. Nat. Immunol. 2019; 20:1372–1380. 31451789 10.1038/s41590-019-0471-5 PMC6754753

[90] Sinha S., van Nimwegen E., Siggia E.D. A probabilistic method to detect regulatory modules. Bioinformatics. 2003; 19(Suppl. 1):i292–i301. 12855472 10.1093/bioinformatics/btg1040

[91] Hoffman M.M., Birney E. An effective model for natural selection in promoters. Genome Res. 2010; 20:685–692. 20194951 10.1101/gr.096719.109 PMC2860170

[92] Wu J., Xie J. Bang H, Zhou XK, van Epps HL, Mazumdar M Hidden Markov model and its applications in motif findings. Statistical Methods in Molecular Biology, Methods in Molecular Biology. 2010; 620:Totowa, NJ Humana Press 405–416. 10.1007/978-1-60761-580-4_13 20652513

[93] Kelley D.R., Snoek J., Rinn J.L. Basset: learning the regulatory code of the accessible genome with deep convolutional neural networks. Genome Res. 2016; 26:990–999. 27197224 10.1101/gr.200535.115 PMC4937568

[94] Kelley D.R., Reshef Y.A., Bileschi M., Belanger D., McLean C.Y., Snoek J. Sequential regulatory activity prediction across chromosomes with convolutional neural networks. Genome Res. 2018; 28:739–750. 29588361 10.1101/gr.227819.117 PMC5932613

[95] Wang M., Tai C., E W., Wei L. DeFine: deep convolutional neural networks accurately quantify intensities of transcription factor–DNA binding and facilitate evaluation of functional non-coding variants. Nucleic Acids Res. 2018; 46:e69. 29617928 10.1093/nar/gky215 PMC6009584

[96] Liu Y., Barr K., Reinitz J. Fully interpretable deep learning model of transcriptional control. 2019; bioRxiv doi:31 May 2019, preprint: not peer reviewed 10.1101/655639. PMC7355248 32657418

[97] Shi W., Fornes O., Wasserman W.W. Gene expression models based on transcription factor binding events confer insight into functional cis-regulatory variants. Bioinformatics. 2019; 35:2610–2617. 30541050 10.1093/bioinformatics/bty992 PMC6662294

[98] Pickrell J.K. Joint analysis of functional genomic data and genome-wide association studies of 18 human traits. Am. J. Hum. Genet. 2014; 94:559–573. 24702953 10.1016/j.ajhg.2014.03.004 PMC3980523

[99] Wen X., Luca F., Pique-Regi R. Cross-population joint analysis of eQTLs: fine mapping and functional annotation. PLoS Genet. 2015; 11:e1005176. 25906321 10.1371/journal.pgen.1005176 PMC4408026

[100] Cannavò E., Khoueiry P., Garfield D.A., Geeleher P., Zichner T., Gustafson E.H., Ciglar L., Korbel J.O., Furlong E.E.M. Shadow enhancers are pervasive features of developmental regulatory networks. Curr. Biol. 2016; 26:38–51. 26687625 10.1016/j.cub.2015.11.034 PMC4712172

[101] Barolo S. Shadow enhancers: frequently asked questions about distributed cis-regulatory information and enhancer redundancy. Bioessays. 2012; 34:135–141. 22083793 10.1002/bies.201100121 PMC3517143

[102] UK10K Consortium Walter K., Min J.L., Huang J., Crooks L., Memari Y., McCarthy S., Perry J.R.B., Xu C., Futema M. et al. The UK10K project identifies rare variants in health and disease. Nature. 2015; 526:82–90. 26367797 10.1038/nature14962 PMC4773891

[103] Sudlow C., Gallacher J., Allen N., Beral V., Burton P., Danesh J., Downey P., Elliott P., Green J., Landray M. et al. UK biobank: an open access resource for identifying the causes of a wide range of complex diseases of middle and old age. PLoS Med. 2015; 12:e1001779. 25826379 10.1371/journal.pmed.1001779 PMC4380465

[104] Schoenfelder S., Sugar R., Dimond A., Javierre B.-M., Armstrong H., Mifsud B., Dimitrova E., Matheson L., Tavares-Cadete F., Furlan-Magaril M. et al. Polycomb repressive complex PRC1 spatially constrains the mouse embryonic stem cell genome. Nat. Genet. 2015; 47:1179–1186. 26323060 10.1038/ng.3393 PMC4847639

[105] Joshi O., Wang S.-Y., Kuznetsova T., Atlasi Y., Peng T., Fabre P.J., Habibi E., Shaik J., Saeed S., Handoko L. et al. Dynamic reorganization of extremely long-range promoter–promoter interactions between two states of pluripotency. Cell Stem Cell. 2015; 17:748–757. 26637943 10.1016/j.stem.2015.11.010

[106] Li G., Ruan X., Auerbach R.K., Sandhu K.S., Zheng M., Wang P., Poh H.M., Goh Y., Lim J., Zhang J. et al. Extensive promoter-centered chromatin interactions provide a topological basis for transcription regulation. Cell. 2012; 148:84–98. 22265404 10.1016/j.cell.2011.12.014 PMC3339270

[107] Wang Q., Sawyer I.A., Sung M.-H., Sturgill D., Shevtsov S.P., Pegoraro G., Hakim O., Baek S., Hager G.L., Dundr M. Cajal bodies are linked to genome conformation. Nat. Commun. 2016; 7:10966. 26997247 10.1038/ncomms10966 PMC4802181

[108] Nguyen T.A., Jones R.D., Snavely A.R., Pfenning A.R., Kirchner R., Hemberg M., Gray J.M. High-throughput functional comparison of promoter and enhancer activities. Genome Res. 2016; 26:1023–1033. 27311442 10.1101/gr.204834.116 PMC4971761

