# Supplementary material for: Functional effects of variation in transcription factor binding highlight long-range gene regulation by epromoters
Source: Nucleic Acids Res. 2020 Feb 29;48(6):2866–79. doi: 10.1093/nar/gkaa123 (PMC7102942; doi:10.1093/nar/gkaa123)
Supplement: gkaa123_Supplemental_Files [file gkaa123_supplemental_files.zip › Mitchelmore_etal_supplementary_figures.pdf]

Functional effects of variation in transcription factor binding highlight long-range gene regulation by epromoters

Joanna Mitchelmore, Nastasiya Grinberg, Chris Wallace and Mikhail Spivakov

Supplementary Figures S1-S4

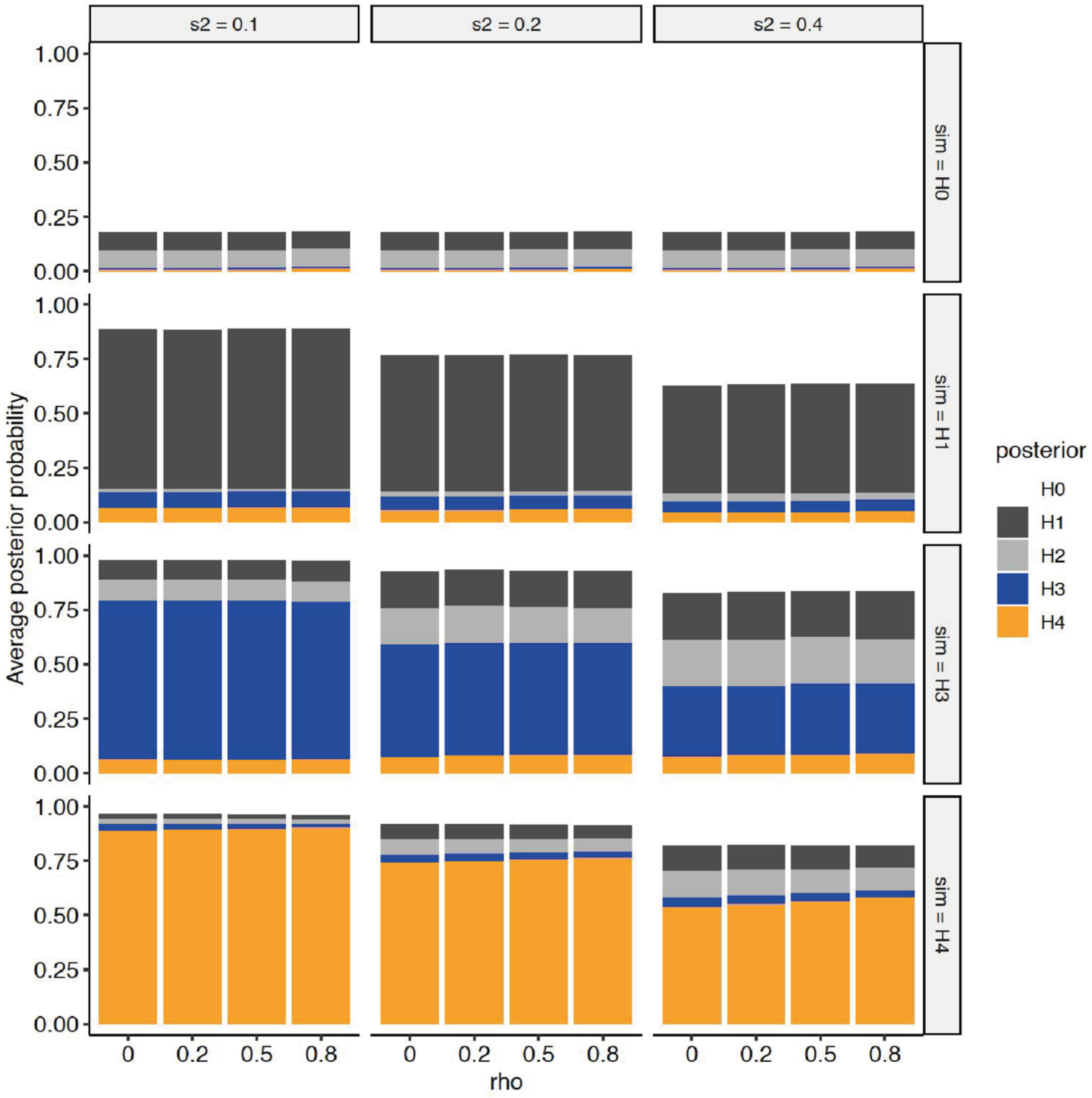

Figure S1. Suitability of the association signal colocalisation algorithm for analysing pairs of signals within the same dataset. We sampled 200 individuals from 1000 Genomes and simulated two quantitative traits with varying levels of non-genetic trait variance (s2, with higher s2 implying lower power) and residual correlation after main genetic effects were accounted for (rho). Genetic effects were simulated as either sim=H0 (no effects for either traits), sim=H1 or sim=H2 (a single causal variant for trait 1 or trait 2, respectively), sim=H3 (different single causal variants for each trait) or sim=H4 (the same single causal variant for both traits). Barplots show the average posterior probability of each of the five possible hypotheses, and show that increasing rho has a negligible impact on inference, reassuring that it is appropriate to use coloc to compare expression of two genes quantified on the same individuals.

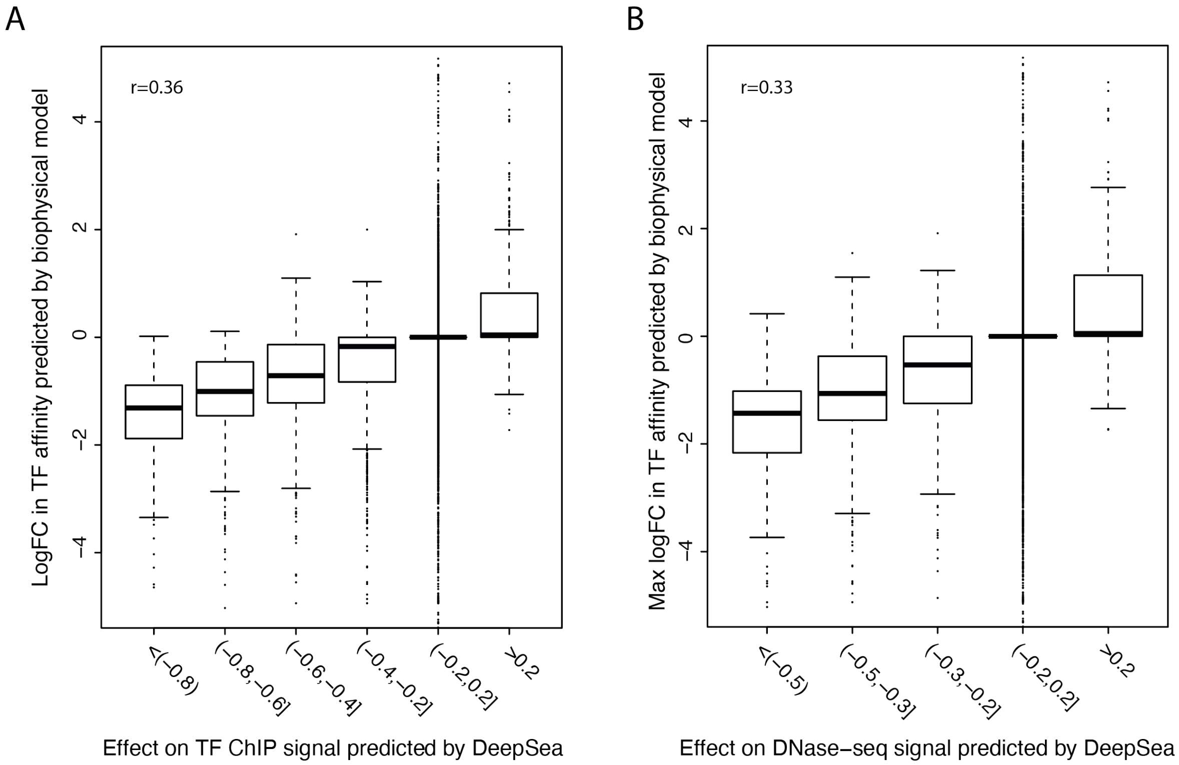

Figure S2. Comparison of SNP effects predicted by the biophysical model used in our study with those predicted by the deep learning model DeepSea. (A) Biophysical model versus DeepSea predictions of SNP effects on TF binding affinity at CRMs for the binding sites of 33 TFs, for which both predictions were available. **(B)** Biophysical model predictions of the maximum effect of a SNP on TF binding at CRMs versus DeepSea predictions of the effect of the same SNP on DNase-seq signal. "Chromatin feature probabilities" are used as a measure of variant effects predicted by DeepSea. Y axes are cropped at (-5;5) for clarity with a loss of some outliers.

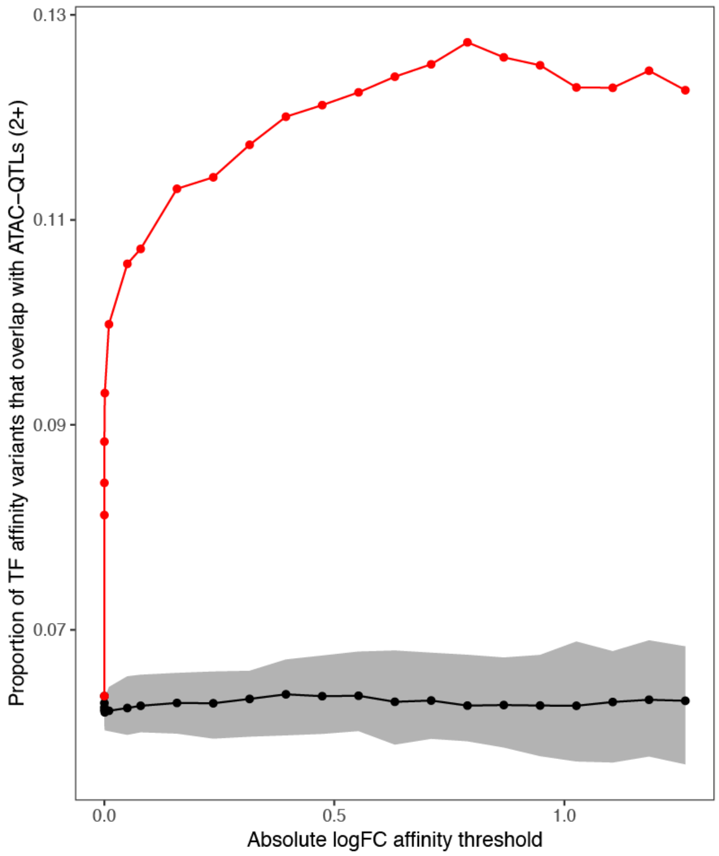

Figure S3. Enrichment of the predicted TF affinity variants for ATAC-QTLs. Proportion of predicted TF affinity variants overlapping with ATAC-QTLs from Tehranchi et al. ($p<0.005$ in 2+ populations) per bins of predicted absolute log-fold change in TF affinity. Red line: observed values, black line: random expectation (median over 100 permutations; grey ribbon: the 0.05-0.95 quantile range). Random expectation was computed by permutation accounting for SNP allele frequencies.

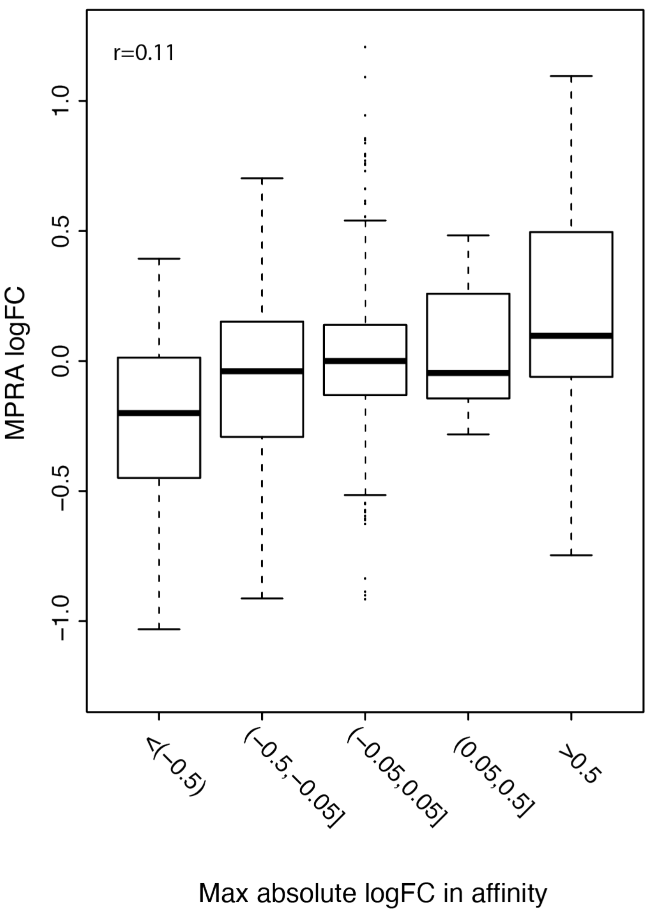

Figure S4. The effects of predicted TF affinity variants on reporter gene expression. Log-skew in MPRA reporter gene expression as a result of ref->alt allele substitution (data from Tewhey et al.) versus the predicted effect of the same SNP on TF binding affinity (maximum effect over all bound TFs is taken).
